# Nutrient Presses and Pulses Differentially Impact Plants, Herbivores, Detritivores and Their Natural Enemies

**DOI:** 10.1371/journal.pone.0043929

**Published:** 2012-08-28

**Authors:** Shannon M. Murphy, Gina M. Wimp, Danny Lewis, Robert F. Denno

**Affiliations:** 1 Department of Biological Sciences, University of Denver, Denver, Colorado, United States of America; 2 Biology Department, Georgetown University, Washington, DC, United States of America; 3 Department of Entomology, University of Maryland, College Park, Maryland, United States of America; University of Alberta, Canada

## Abstract

Anthropogenic nutrient inputs into native ecosystems cause fluctuations in resources that normally limit plant growth, which has important consequences for associated food webs. Such inputs from agricultural and urban habitats into nearby natural systems are increasing globally and can be highly variable, spanning the range from sporadic to continuous. Despite the global increase in anthropogenically-derived nutrient inputs into native ecosystems, the consequences of variation in subsidy duration on native plants and their associated food webs are poorly known. Specifically, while some studies have examined the effects of nutrient subsidies on native ecosystems for a single year (a nutrient pulse), repeated introductions of nutrients across multiple years (a nutrient press) better reflect the persistent nature of anthropogenic nutrient enrichment. We therefore contrasted the effects of a one-year nutrient pulse with a four-year nutrient press on arthropod consumers in two salt marshes. Salt marshes represent an ideal system to address the differential impacts of nutrient pulses and presses on ecosystem and community dynamics because human development and other anthropogenic activities lead to recurrent introductions of nutrients into these natural systems. We found that plant biomass and %N as well as arthropod density fell after the nutrient pulse ended but remained elevated throughout the nutrient press. Notably, higher trophic levels responded more strongly than lower trophic levels to fertilization, and the predator/prey ratio increased each year of the nutrient press, demonstrating that food web responses to anthropogenic nutrient enrichment can take years to fully manifest themselves. Vegetation at the two marshes also exhibited an apparent tradeoff between increasing %N and biomass in response to fertilization. Our research emphasizes the need for long-term, spatially diverse studies of nutrient enrichment in order to understand how variation in the duration of anthropogenic nutrient subsidies affects native ecosystems.

## Introduction

Natural and anthropogenic inputs of nutrients into native ecosystems often promote fluctuations in the availability of resources that normally limit plant growth [1], [2], [3], [4]. Such inputs promote changes in primary productivity and plant diversity that in turn can have important, community-wide consequences for associated food webs [4], [5], [6], [7], [8]. Even a short-term increase in a resource that is limiting (a resource pulse) can have extended effects on community structure, trophic interactions and ecosystem function [9]. Although a growing number of studies document the widespread effects of sporadic nutrient or basal-resource pulses on plant productivity and food web structure [5], [7], [9], [10], [11], [12], [13], [14], [15], [16], [17], [18], few have examined how long-term nutrient loading (a resource press) impacts recipient communities [3], [4], [19], [20], and fewer yet have contrasted the food web effects of a nutrient pulse with a press in the same system [3], [21]. In previous work, we found that increased primary production via nitrogen fertilization alters arthropod community structure and composition in *Spartina* marshes; species richness of herbivores, predators, parasitoids and detritivores all increased in response to nitrogen addition [18]. That study was the first to examine how food web structure is altered through trophic dynamics that extend solely from enhanced plant production, and not from changes in plant community composition, but it only examined the food web response to a nutrient pulse within a single season. What happens to arthropod food webs when wetlands receive a nutrient press over several years is unknown yet of critical importance given the increasing amount of nitrogen runoff into salt marshes. There are a few notable examples of ongoing nutrient press studies [22], [23], [24], [25], [26], [27] but these studies focus primarily on plant responses to nutrient subsidies and the responses of multiple trophic levels to such presses remains poorly understood. Here we extend our previous research on food web responses to nutrient subsidies to compare the effects of a resource pulse with a resource press on the arthropod food web of two mid-Atlantic salt marshes in North America.

Inputs of limiting nutrients (e.g., nitrogen) frequently have important effects on plant species richness, plant community composition, primary productivity, and plant tissue quality [3], [4], [28]. For example, long-term nutrient loading can lead to the simplification of both plant and associated arthropod communities due in part to a strong correlation between plant and insect species diversity [4]. As a result, determining the direct effects of enrichment on arthropod communities, as opposed to indirect effects mediated by plant composition, can be experimentally daunting [3], [4], [19], [21]. However, by restricting our research to the natural monocultures of *Spartina alterniflora* (hereafter *Spartina*) found in salt marshes, our study is uniquely able to focus on altered food web structure and dynamics that extend solely from enhanced plant productivity and not from compositional changes in the plant community [13].

Understanding how the duration of anthropogenically-derived nutrient subsidies affects natural food webs is important from a conservation perspective in light of the nearly seven-fold increase in agricultural nitrogen fertilization and an extreme global increase of nutrient runoff into natural systems [29]. In particular, land development and agriculture are jeopardizing coastal wetlands at an alarming rate, and one of the major threats is nutrient pollution from neighboring anthropogenic sources, which alters vegetation dynamics by increasing nitrogen availability [30], [31]. An estimated 50% of the variation in nitrogen availability in *Spartina* marshes is explained by shoreline development, such as housing developments and agriculture [30]. Annual nitrogen inputs from fertilizer exceed 1000 kg/km^2^ and are expected to double in the near future [32], [33], [34]. In addition to fertilizer-derived nitrate, animal waste is estimated to add more than 2000 kg/km^2^ and waste water from urban areas contributes an additional 100–500 kg/km^2^ to the annual nitrogen-load in aqueous runoff [35], [36]. As terrestrially-derived nitrate flows downstream, about one quarter is intercepted by coastal wetlands (e.g. *Spartina* marshes) before reaching open waters [37]. Nitrogen that is retained in the marsh is incorporated into plant biomass, denitrified or buried in marsh sediments [13], [37], [38]. Because *Spartina* is N-limited, nitrogen subsidies result in dramatic increases in biomass, plant nitrogen content and detritus [13], [14], [30], [39], [40]. Stable isotope analyses confirm that allochthonous nitrogen is taken up by *Spartina* [41] and is transported directly up the food chain from producers to primary consumers [42]. Thus, nutrient runoff from anthropogenic sources has direct consequences for *Spartina* and its associated consumers. Our research on how nutrient pulses and presses alter food web structure is particularly relevant because inputs of nutrients from agricultural and urban habitats into nearby natural systems can be highly variable and span the range from sporadic (e.g. nutrient pulse) to continuous (e.g. nutrient press) [32], [35], [37], [43]. Yet, how nitrogen pulses and presses differentially affect the recipient community of consumers is poorly known.

Some arthropod species may respond to a nutrient press by retaining the density achieved during the first year of enrichment, but others are likely to exhibit more complex responses. Enrichment affects each arthropod species indirectly via one or more paths through the complex marsh interaction web. Feedbacks and time lags may mean that the effect of enrichment on a species may continue to change over many years. For example, increased live plant biomass can be expected to produce increased thatch (dead) biomass after a time lag, which is in turn expected to decrease intraguild predation and cannibalism [44], [45]. An increase in predator population growth rate may follow, with subsequent effects on prey density. Such interactions among responses may continue to reverberate over long time frames and are not predictable from short-term experiments.

Here, we present the results of an experiment in which we tested the responses of the arthropod food web to a nutrient pulse and a nutrient press. We replicated our experiment at two field sites, Tuckerton, NJ (TUCK) and Cape Hatteras National Seashore, NC (CHNS), which are located on opposite sides of a biogeographic break in VA where *Spartina* switches from annual to perennial aboveground growth [46]. We chose to work in a higher-latitude marsh and a lower-latitude marsh to investigate site-to-site variation in pulse and press dynamics. Pennings *et al.* [47] showed that *Spartina* from marshes at higher latitudes in North America is more palatable to herbivores than in marshes at lower latitudes. Recently, McCall and Pennings [48] demonstrated that latitude and tidal range explain much of the geographic variation in biotic and abiotic variables among marshes at higher and lower latitudes. Notably, the two marshes examined in this study vary greatly in tidal range (longer tidal inundation at CHNS relative to TUCK), which is positively correlated with *Spartina* height [49]. The differences in our study marshes may play an important role in how readily plants use nutrient subsidies and the degree to which the arthropod community responds.

**Figure 1:**
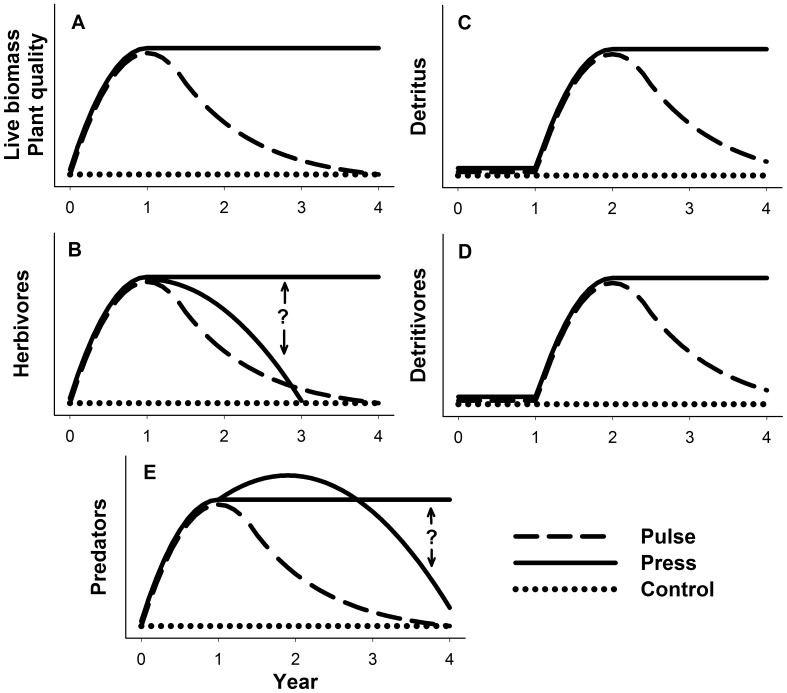
Conceptual model of possible inter-annual responses of *Spartina* plant parameters and consumer densities to press and pulse nitrogen subsidies. Plant characteristics (A, C) and arthropod densities (B, D, E) are expected to vary over time due to seasonal and stochastic events not related to the treatment effect. Here we show the predicted responses of the pulse and press treatments in relation to the control treatment, which is held constant over time. Question mark (?) in B represents possible different responses by herbivores depending on level of predator suppression. Question mark (?) in E represents possible different responses by predators depending on whether predators over-exploit their prey.

We expected our nutrient pulse to produce results similar to those of earlier marsh pulse fertilization experiments [13], [14], [15], [18]. In pulse plots, we predicted that 1) species at all trophic levels would respond positively to enrichment, although the increase in detritus and detritivores would be delayed, 2) effects would be greater among higher trophic levels, and 3) all responses would gradually return to control levels after fertilization ceased (Fig. 1). In press plots, which received fertilizer over multiple years, we predicted that herbivores and predators might exhibit complex extended responses (indicated by ‘?’ in Fig. 1b,e). Notably, we expected that a nutrient press would not be a simple extension of a nutrient pulse due to effects on higher trophic levels that could cascade to affect herbivorous prey. Reproductive responses by univoltine spiders to increased prey could lead to increased predator density during the second year, and could be further amplified by an increase in thatch, which reduces predator interference [45]. These increased predator densities were expected to depress herbivore densities [50], but if suppression was relatively mild, herbivore densities could remain elevated and allow predator densities to continue to rise. In contrast, more intense predation could lead to a severe decline in herbivores, followed by a decline in predators. Similar feedbacks and delayed responses may occur among plants, detritus and detritivores, but since those dynamics have been studied less, we predicted only that these groups would retain the levels reached during the first year of fertilization (Fig. 1a,b,d). Finally, we predicted that some responses to fertilization would differ between the two marshes. We predicted that herbivores and predators would respond more strongly to fertilization at TUCK, the high-latitude marsh, because of expected higher grass palatability [47]. We also predicted that *Spartina* height would increase more at CHNS, where tidal inundation was greater.

## Materials and Methods

### Study Sites and Organisms

We conducted our study in two salt marshes dominated by natural monocultures of *Spartina* along the east coast of the United States, TUCK (39° 31.6′N, 74° 19.2′W) and CHNS (35° 47.6′N, 75° 32.8′W). All necessary permits were obtained for the described field studies (permits for TUCK were obtained from K. Able at the Rutgers University Marine Station and permits for CHNS were obtained from B. Commins at the National Park Service Research Permit and Reporting System and J. Ebert, M. Lyons, T. Broili, M. Carfioli, and S. Strickland at CHNS). We focused on a reduced food web composed of the numerically-dominant herbivores, detritivores, and predators that are common at both field sites [18]. *Spartina* serves as the only host plant for a variety of insect herbivores [13]. Planthoppers (*Prokelisia dolus* and *P. marginata*) are most abundant (∼80% of herbivore biomass); remaining herbivores are rare relative to *Prokelisia* and consist of other planthoppers (e.g., *Delphacodes penedetecta*) and true bugs (e.g., *Trigonotylus uhleri*). Detritivores feed on *Spartina* detritus and epiphyton associated with *Spartina* on the marsh surface (e.g. amphipod *Orchestia grillus*, isopod *Venezillo parvus*). Natural enemies, including invertebrate predators and parasitoids, attack herbivores and detritivores associated with *Spartina*, but predators are the most important source of mortality [50]; these include omnivores that feed on *Spartina* and other herbivores (e.g. katydids *Conocephalus spartinae* and *Orchelimum fidicinium*), generalist predators (e.g. web-building spider *Grammonota trivittata*), and specialist predators (e.g. the mirid *Tytthus vagus* attacks planthopper eggs) [44]. Top carnivores (e.g. hunting spiders *Pardosa littoralis*, *Clubiona* sp.) feed on herbivores, detritivores, specialist predators and sometimes each other [51]. All organisms are hereafter referred to by their genera or feeding guild.

### Nutrient Manipulations

To investigate the differential effects of a nutrient pulse versus press on the arthropod food web, we manipulated nutrient subsidies with a one-way design. Year 1 (2005) was the only year that we fertilized pulse treatment plots. In years 2–4 (2006–2008), we continued to fertilize press plots in the same manner as during year 1 (see Table S1 for a complete list of fertilizer addition and sample dates). At each site, we established 10 blocks, each with three 2×2 m treatment plots, and assigned plots randomly to one of three treatments: control (no fertilization), pulse (fertilization during year 1 only) and press (fertilization during all years). Our plots were necessarily 2×2 m to accommodate restrictions associated with working in a protected National Seashore (CHNS), but previous work shows that the population dynamics of the major herbivores and predators as well as treatment effects on trophic composition in our plots scale up to the dynamics that prevail in larger plots (>100 m^2^) [13], [50]. We fertilized each plot with 60 grams/m^2^ of a 3∶1 mixture of granular ammonium nitrate (N-P-K: 34-0-0) and triple phosphate (0-45-0) three times during the season, for a total of 180 grams/m^2^ per year (Table S1). The elevated N-content achieved by our fertilization treatment is comparable to that for plants in *Spartina* marshes that experience high nutrient loading from nearby coastal developments [30]. Previous work has demonstrated that N additions after peak biomass is attained have little impact on *Spartina* growth [52], thus we applied N only at the beginning of each growing-season for all fertilization treatments.

### Plant and Arthropod Samples

We measured *Spartina* biomass and height before the initiation of fertilization treatments (the first sample collected in May 2005) and subsequently during peak biomass each season (Table S1). On each collection date, we harvested all of the plant biomass within a 0.047-m^2^ quadrat from each plot. We sorted quadrat samples into live and dead (thatch) plant material, measured the height of living culms and counted the number of tillers. We washed the plant material with deionized water, dried it in a drying oven at 60°C for three days and weighed it. To measure the treatment effects on the N-and C-content of *Spartina*, we collected plant snips (5–10 *Spartina* culms per plot) that were processed as described above, ground in a Wiley mill, and sent to the Cornell Stable Isotope Laboratory for analysis. Plant snips and quadrats were collected several times during each growing season (Table S1). To ensure that our fertilization treatments remained in the appropriate treatment plot and did not spread into the adjacent matrix or neighboring plots, we collected plant snips from 1 m outside each plot in 2005 and 2006 (Table S1).

To measure the treatment effects on the arthropod food web, we sampled arthropods with a D-vac suction sampler with a restricted suction head (0.036 m^2^), which we placed in 5 different locations within each plot for 3-seconds. At both sites, we sampled the arthropod community 5 times in 2005 and 2006, and 4 times in 2007 and 2008 (Table S1). After collection, we stored the arthropod samples in ethanol and later sorted, counted and identified individuals to genus and species.

We expected plant characteristics and arthropod densities to vary over time, even in control plots; therefore, to isolate the effects of fertilization from other types of variation, we calculated pulse and press treatment effects in each block, based on the treatment/control ratio. More precisely, treatment effect  =  ln((treatment value +1)/(control value +1)), where “treatment value” was the value from the press or pulse plot in the block, and “control value” was the value from the control plot in the same block (units in g/m^2^ for plant characteristics and individuals/m^2^ for arthropods). A positive treatment effect therefore means that the value in the pulse or press plot was higher than the value in the control plot in the same block.

### Herbivore Damage

To assess the amount of damage inflicted by herbivores, we haphazardly chose 10 *Spartina* culms from each quadrat from CHNS on August 17, 2006. We were able to distinguish damage by three different herbivores: snail, katydid and *Trigonotylus*. For each leaf, we measured length and the amount of leaf that was damaged to the nearest 0.5 cm; for snails we measured the length of each radulation, for katydids we measured the length of each chew mark and for *Trigonotylus* we measured their distinctive ‘spotting’ damage. Snail damage was minimal so we did not include it in our analyses. We then divided each type of damage by the total length of all leaves in the culm to get average damage per cm of leaf.

### Statistical Analyses

To test for pre-treatment differences, we performed ANOVA on plant and arthropod characteristics in the May 2005 pre-treatment sample, using the treatment that a plot would later receive (press, pulse or control) as the explanatory variable (SAS proc anova). For subsequent statistical analyses, we calculated the effect of fertilization treatment on each *Spartina* characteristic and on the density of each arthropod taxon in each block on each sample date. We used treatment effect as the response variable in a repeated measures ANOVA with explanatory variables time, marsh (CHNS or TUCK), treatment (press or pulse) and all possible interactions (SAS proc mixed). Block within marsh was treated as a random effect. We treated pre-fertilization data collected in May 2005 as a separate time category from the post-fertilization data collected later the same year. In order to determine the appropriate variance-covariance structure for our repeated measures ANOVA, we first explored temporal autocorrelation among data. We used an autoregressive structure when correlation decayed over time (AR(1) in proc mixed), compound symmetry (CS in proc mixed) when correlation showed no temporal trend, and a simple variance components matrix (VC in proc mixed) when autocorrelation was absent. When variance changed over time, we used a heterogeneous structure (ARH(1) or CSH in proc mixed). When there was a significant year by treatment interaction, we used pre-planned t-tests to compare the effects of press and pulse treatments on plant characteristics and arthropod densities at both marshes during each of the four years and during the pre-treatment collection. A false error rate correction for multiple tests was applied to the results of those tests (SAS proc multtest). To test whether fertilizer remained in the appropriate treatment plot and did not spread into the adjacent matrix or neighboring plots, we performed ANOVA on live *Spartina* biomass and culm height 1 m outside plots in 2005 and 2006; treatment was the fixed effect and block and marsh were random effects. We calculated herbivore load for each plot on each sample date as (herbivore density +1)/(*Spartina* live biomass). Herbivore density was the density of all planthoppers, katydids and *Trigonotylus*. We then calculated the effect of press and pulse treatments on herbivore load in each block on each sample date as ln(treatment load/control load). In a similar manner, we calculated predator/prey ratio for each plot as (predator density +1)/(prey density +1), and then calculated effect sizes as above. Predators consisted of all spiders and prey consisted of the herbivores listed above. As a measure of total nitrogen uptake by *Spartina*, we used grams nitrogen in live biomass per square meter of marsh surface, calculated as %N multiplied by live biomass per square meter. We tested for differences in this N density between marshes and fertilization treatments with repeated measures ANOVA (SAS proc mixed) using marsh, fertilization treatment and their interaction as fixed factors and block within marsh as a random effect. For herbivore damage, we analyzed *Trigonotylus* damage using repeated measures ANOVA with block as a random effect and the ten culms treated as repeated measures from the same plot (SAS proc mixed). Katydid-damaged area had so many zero values that parametric analysis was not possible, so we used the randomization test of Ruxton et al. [53], modified to account for repeated measures [54]. Separate tests were performed on all treatment pairs and P-values were adjusted for multiple tests (SAS proc multtest). We deposited our data in the Dryad Repository: http://dx.doi.org/10.5061/dryad.fb006 [55].

## Results

**Figure 2:**
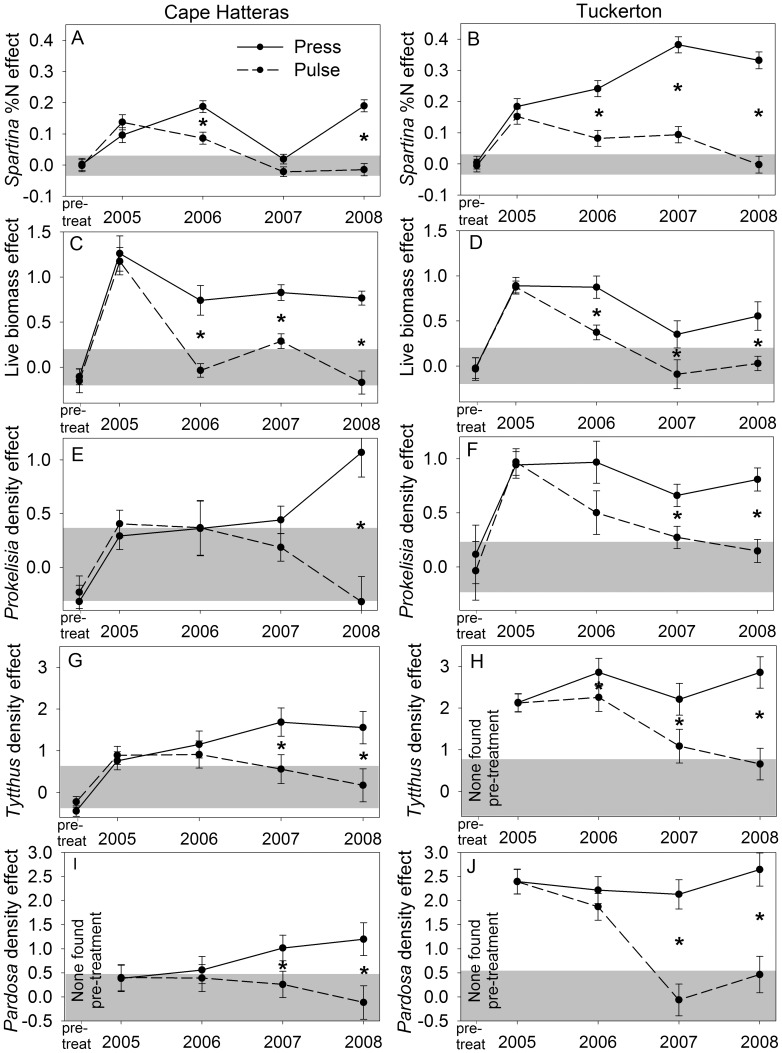
Plant and arthropod responses to nutrient manipulations. Effects of press (solid line) and pulse (dashed line) treatments on *Spartina* %N (A, B), *Spartina* live biomass density (C, D), density of *Prokelisia* planthoppers (E, F), density of the predatory mirid *Tytthus* (G, H), and density of the top intraguild predator *Pardosa* (I, J) (results from CHNS on left, TUCK on right; means ±se). Effect means within gray bands were not significantly different from zero, meaning that treatment and control values did not differ. Asterisks indicate that pulse and press treatments had significantly different effects in a given year (α. = 0.05). Asterisks are displayed only when the year by treatment interaction was significant. See methods for information on how treatment effects were calculated.

With some notable exceptions, the 19 plant and arthropod response variables followed similar trajectories over the course of the experiment. In plots that received the one-year fertilizer pulse, responses were significantly greater than controls during the first year, after which they gradually declined, and by year 4 they were no different from controls (Figs. 2, S1, S2, S3). These results replicated those of earlier pulse experiments. In plots that received press fertilization, on the other hand, responses remained higher than controls throughout the four years of the experiment.

### Pre-treatment Differences and Fertilization Effects Outside Study Plots

In our pre-treatment sample, treatments did not differ significantly from each other for any measures, except at CHNS where *Tytthus* densities were lower in plots that would later receive nutrient presses than in control plots (*F*_2,27_ = 3.80, *P* = 0.03). We detected no effect of fertilization treatment on *Spartina* biomass (year 1: *F*_2,46.1_ = 0.32, *P* = 0.73; year 2: *F*_2,46.3_ = 0.51, *P* = 0.60), or on culm height 1 m outside of treatment plots (year 1: *F*_2,47_ = 0.21, *P* = 0.81; year 2: *F*_2,46.3_ = 0.56, *P* = 0.58).

### Plant Responses to Nutrient Manipulations

**Figure 3:**
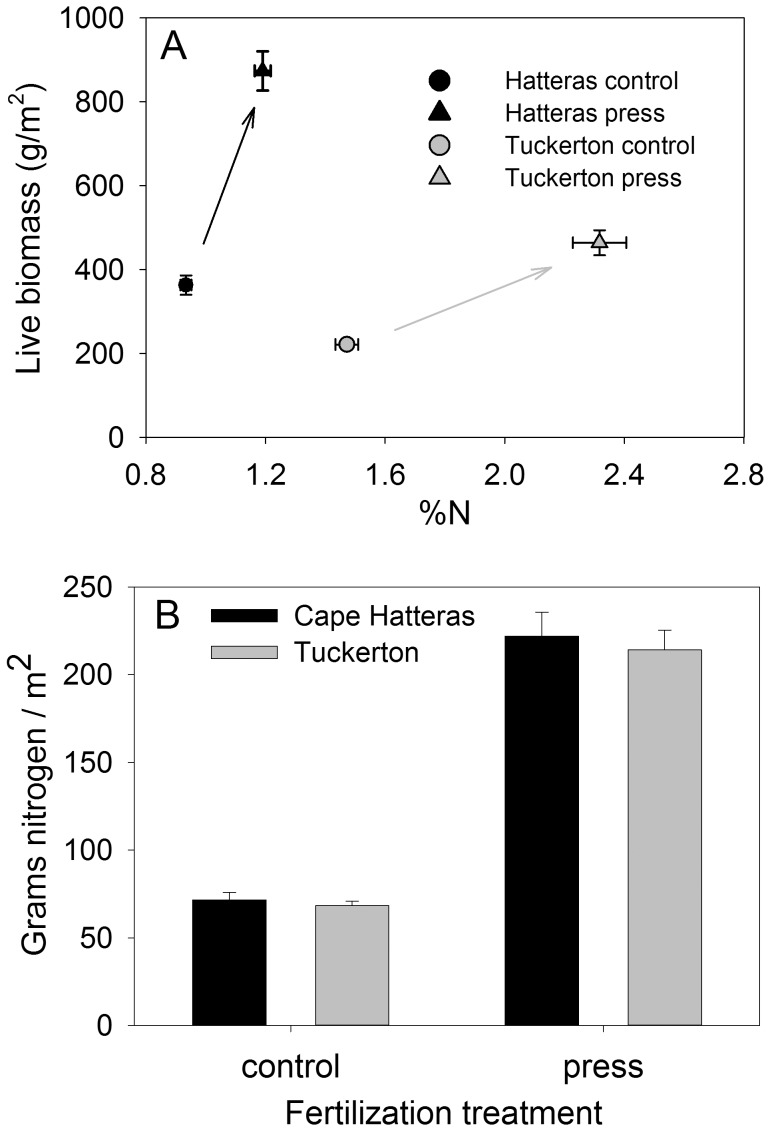
Similarities and differences in the *Spartina* response to fertilization at CHNS and TUCK (means ±se). Responses of *Spartina* live biomass and %N to press fertilization differed significantly between the two marshes when averaged over the duration of the study (A). Arrows indicate the change caused by fertilization at each marsh. In contrast, fertilization increased nitrogen density (gN/m^2^ in live *Spartina*) by the same amount at the two marshes when averaged over the duration of the study (B).

*Spartina* %N increased in response to fertilization, but it responded much more strongly at TUCK than at CHNS (marsh*treatment interaction *F*_1,28.7_ = 40.37, *P*<0.0001) (Fig. 2a,b). In contrast, live *Spartina* biomass (measured as dry weight) responded more strongly at CHNS (marsh*treatment interaction *F*_1,65.5_ = 12.48, *P* = 0.0008) (Fig. 2c,d). These responses exaggerated already-existing differences between control plots at the two marshes, where percent nitrogen was higher at TUCK (*t*_29.9_ = 17.50, *P*<0.0001), and live biomass was higher at CHNS (*t*_6.31_ = 2.69, *P* = 0.03). As a result, the two marshes became even more different from one another when fertilized (Fig. 3a). Despite these significant differences between marshes, *Spartina* nitrogen density (grams N in live Spartina/m2) in control plots was very similar at the two marshes (*t*_80.4_ = 0.64, *P* = 0.52) and increased by virtually the same, very large, amount when fertilized (Fig. 3b). Nitrogen density in press treatments did not differ between marshes (*t*_76.6_ = 0.45, *P* = 0.65), but was higher than control plots (*t*_88.1_ = 16.14, *P*<0.0001). At CHNS this result was accomplished largely through increased biomass whereas at TUCK this happened largely through increased %N with a relatively small increase in biomass.

The response of thatch to fertilization was delayed by a year, but starting in year 2 it followed the usual trajectory: thatch in pulse plots peaked in year 2 and then declined, whereas thatch in press plots remained elevated through year 4 (Fig. S1e). However, it is notable that thatch levels in press plots declined in years 3 and 4 even though they remained above control levels.

Contrary to our prediction, culm length responded more strongly at TUCK than at CHNS (*F*_1,19.6_ = 6.40, *P* = 0.02) (Fig. S1a), despite the fact that tidal inundation was greater at CHNS. Culm density exhibited a positive response to fertilization only during year 1 (Fig. S1b). Tiller density was not affected by fertilization at either marsh (*F*_1,102_ = 0.09, *P* = 0.77) (Fig. S1c).

### Arthropod Responses to Nutrient Manipulations

Densities of arthropods exhibited positive responses to pulse and press fertilization with few exceptions (Figs. 2, S2, S3). Those exceptions included densities of the amphipod *Orchestia*, which responded erratically to fertilization, increasing and decreasing in different years (Fig. S2c). *Prokelisia* planthopper density responded positively at TUCK, but did not become significantly greater than controls in CHNS press plots until year 3 (Fig. 2e,f). Finally, in press plots at TUCK, densities of *Trigonotylus* (Fig. S2b) and hunting spiders (Fig. S3c) were not significantly greater than controls in year 3.

**Figure 4:**
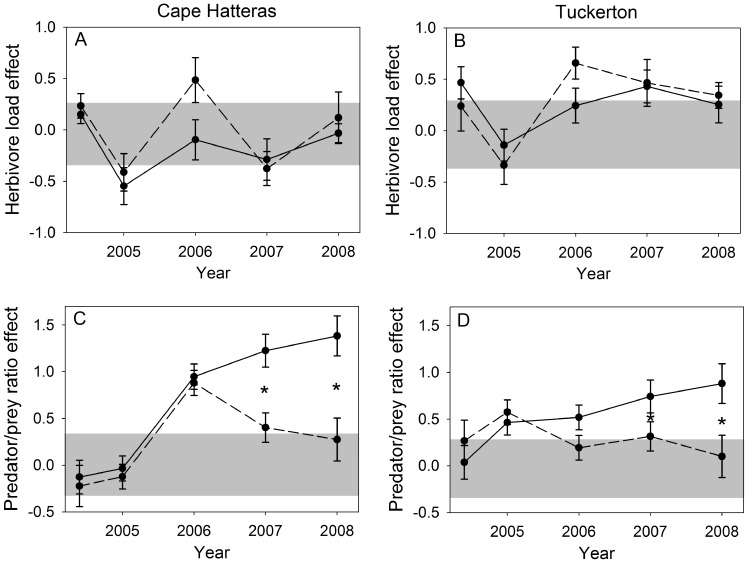
Response of herbivore load and predator/prey ratio to nutrient manipulations. Effects of press (solid line) and pulse (dashed line) treatments on herbivore load (A, B) and predator/prey ratio (C, D) at the CHNS and TUCK (means ±se). Effects within gray bars were not significantly different from zero, meaning that treatment and control plots did not differ. Herbivores consisted of all planthopper adults and nymphs plus *Trigonotylus*; predators consisted of all spiders. Asterisks indicate that pulse and press treatments had significantly different effects in a given year (α = 0.05).

Under press fertilization, most predator densities remained elevated (Figs. 2g–j, S3b,c) and the predator/prey ratio increased throughout the experiment at both marshes (Fig. 4c,d). However, the resulting increase in predation pressure was not sufficient to depress herbivore densities, all of which were greater in the press than control during the last year of the experiment at both marshes (Figs. 2e,f, S2a,b).

Five of the nine arthropod response variables that were measured at both marshes differed significantly between marshes, and all five responded more strongly at TUCK than at CHNS, supporting our prediction of stronger responses at the high-latitude marsh, possibly due to more palatable grass. Those five responses were densities of *Prokelisia* planthoppers (*F*_1,2.2_ = 9.64, *P* = 0.0051) (Fig. 2e,f), *Delphacodes* planthoppers (*F*_1,20.4_ = 33.26, *P*<0.0001) (Fig. S2a), *Tytthus* egg predators (*F*_1,44.1_ = 24.78, *P*<0.0001) (Fig. 2g,h), *Pardosa* wolf spiders (*F*_1,20.4_ = 35.13, *P*<0.0001) (Fig. 2i,j), and herbivore load (*F*_1,9.92_ = 9.25, *P* = 0.013) (Fig. 4a,b).

Although herbivore load was significantly higher at TUCK than at CHNS, fertilization had a relatively weak effect on herbivore load even at TUCK (*t*_15.1_ = 1.76, *P* = 0.099) (Fig. 4a,b), indicating that herbivore density increased by roughly the same percentage as *Spartina* biomass in response to fertilization. The fraction of *Spartina* tissue damaged by katydids increased with fertilization (*P*<0.0001; Fig. S4a), and damage in the press treatment was greater than the pulse treatment (P = 0.02; Fig. S4a). In contrast, fertilization did not reliably increase damage by *Trigonotylus*; pulse plots had significantly higher levels of damage than controls (*t*_22.9_ = 3.56, *P* = 0.0017), but press plots did not (*t*_22.9_ = 0.03, *P* = 0.98) (Fig. S4b).

## Discussion

As in earlier studies of short-term nutrient pulses [14], we found that plants and arthropods on both marshes responded positively to a nutrient pulse during the first year and then gradually returned to control levels over the next four years. When nutrient addition was maintained over multiple years as a nutrient press, many responses simply maintained the level reached during the first year of fertilization, but others exhibited more complex, extended responses. As predicted, the responses by higher trophic levels to the extended fertilization of a nutrient press meant that results from a pulse study could not have predicted food web responses to a nutrient press. One key food web characteristic, the predator-to-prey ratio, continued to increase in press plots over the course of the experiment at both marshes (Fig. 4c,d). Thatch biomass, which has important effects on predator interference and prey suppression [45], showed the opposite trend. Thatch peaked during the second year of fertilization and then declined at both marshes even under continued fertilization (Fig. S1e). The fact that these extended responses occurred in a natural monoculture shows that long-term enrichment can affect predator-prey interactions without the mediation of plant species turnover, and that long-term experiments are valuable in determining extended effects.

Predator densities generally responded more robustly to enrichment than herbivore densities, increasing the predator/prey ratio in agreement with theory and earlier pulse fertilization experiments [13], [15], [56]. However, the continued rise of the predator/prey ratio in press treatments relative to controls contrasts with earlier pulse studies and may have been caused by slow-developing predator responses, including reproductive responses, and their response to increased thatch and *Spartina* biomass. Web-building spiders in particular may have benefitted from more space for web construction. Marsh predators, especially *Tytthus* and *Pardosa,* are capable of suppressing herbivore densities [45], and our predictions included the possibility that increased predator abundance would lead to over-exploitation of prey and subsequent decline in predator densities (Fig. 1b,e). However, it appears that improved *Spartina* quantity and quality allowed herbivore abundance to increase despite increased predation pressure (Figs. 2e,f, S2a,b). The decrease in thatch biomass in press plots relative to controls during years 3 and 4, after a peak in year 2 (Fig. S1e) may have been caused by a delayed response of decomposers to increased thatch quality [57]. Because higher levels of thatch decrease cannibalism, this trend toward lower thatch levels may eventually lead to an increase in intraguild predation and cannibalism, potentially reducing herbivore suppression and increasing herbivore damage. Such feedback loops highlight the importance of conducting nutrient press studies because the effects of nutrient addition on higher trophic levels, and thereby trophic cascades, may take years to be fully manifested.

In addition to differences between nutrient pulses and presses, the contrast in how *Spartina* responded to a nutrient press at the two marshes was striking. At TUCK, plant quality (%N) increased with a minimal increase in biomass, while at CHNS biomass increased with only a small increase in quality (Fig. 3). Our results demonstrate that vegetation at different sites can respond to the same degree of enrichment in very different ways, perhaps due to a tradeoff between increases in plant quality and biomass. We found that the arthropod food web had a greater response to nutrient subsidies at TUCK than at CHNS, perhaps because consumers responded more readily to increases in plant quality than biomass. The modest increase in plant quality at CHNS may explain why herbivores took longer to respond than at TUCK and why there was a much weaker effect on higher trophic levels. Our prediction that detritus (thatch) would not accumulate until the second year of the experiment, was supported at both sites (Fig. S1e). However, detritivores did not always track the detrital signal. *Orchestia* demonstrated positive, negative, or neutral responses to fertilization depending on year (Fig. S2c). *Venezillo* responded positively to fertilization, with a stronger response to the nutrient press (Fig. S2d). Although the overall response of *Venezillo* met predictions, densities were not lagged as predicted. Both of these species have been recorded in the literature as feeding on *Spartina* detritus [58], [59], but may also feed heavily on live *Spartina* or algae [60], [61], which may explain why their responses did not correspond with our predictions for ‘true’ detritivore species.

Herbivore responses varied spatially between marshes; the magnitude and duration of planthopper responses were both greater at TUCK (Figs. 2e,f, S2a), but the herbivore *Trigonotylus* had a greater response to the press treatment at CHNS (Fig. S2b). Although herbivore densities increased in pulse and press plots as predicted, our prediction that we would also observe greater levels of herbivore damage on *Spartina* plants in those plots was mixed. Katydid densities and damage were greater in pulse and press plots relative to controls (Figs. S3a, S4a), but greater *Trigonotylus* densities did not translate into increased damage in pulse and press treatments (Figs. S2b, S4b).

Our study demonstrates that nutrient subsidies can have very different impacts on the arthropod food web depending on subsidy duration. In general, plant and arthropod measures returned to ambient conditions within 3–4 years after a nutrient pulse, but remained elevated during the entirety of a nutrient press. Several observed responses displayed consistent trends as the press continued, including increases in the predator/prey ratio, densities of *Pardosa* and web-building spiders, and a decrease in thatch biomass after the spike in year 2. These long-term trends imply that the ultimate effect of enrichment on these marshes remains unknown. Nutrient pulses and presses can also have very distinct responses in different salt marshes; in the high-latitude marsh, plants responded by increasing plant quality while in the low-latitude marsh, plants increased biomass. Arthropod response was more consistent between marshes, but the magnitude of response was much greater at TUCK, the high-latitude marsh, perhaps due to individual taxa responses to higher plant quality (%N) at that marsh.

Recently there has been a call for long-term studies on resource pulses and their direct and indirect effects on the recipient plant and arthropod communities [62], [63]. Our research suggests that investigations into how resource pulses and presses differ is also necessary, especially in systems where input regimes of nutrients from anthropogenic sources into natural systems, such as salt marshes, can be highly variable. As agricultural production and nitrogen application continues to intensify, natural ecosystems will experience a long-term press in nitrogen loading from anthropogenic sources. Our study demonstrates that persistent nitrogen addition has the potential to reshape food web interactions by differentially impacting higher trophic levels. While previous nutrient press studies have primarily focused on the impacts of nutrient addition on plants and herbivores, here we demonstrate that nutrient additions may lead to feedback loops that impact prey suppression and ultimately change rates of plant production and decomposition via increasingly greater impacts on natural enemies.

## Supporting Information

Figure S1 **Effects of press and pulse treatments on additional** ***Spartina*** **characteristics not included in** **Figure 1** **.** Effects for press (solid line) and pulse (dashed line) treatments are displayed for Cape Hatteras National Seashore, NC (panels on left), and Tuckerton, NJ (panels on right). Error bars indicate standard errors of the means. Effect means within gray bands were not significantly different from zero, meaning that treatment and control values did not differ. Asterisks indicate that pulse and press treatments had significantly different effects in a given year (alpha  = 0.05). Treatment effect was calculated as ln((treatment value +1)/(control value +1)), where “treatment value” was the value from the press or pulse plot in a block, and “control value” was the value from the control plot in that same block. A) Effect of press (solid line) and pulse (dashed line) treatments on the average length of *Spartina* culms (plant height). B) Effect of press (solid line) and pulse (dashed line) treatments on the density of *Spartina* culms per square meter. Neither fertilization treatment had a consistent affect on culm density at either marsh. C) Effect of press (solid line) and pulse (dashed line) treatments on the number of *Spartina* tillers per square meter. Neither fertilization treatment significantly affected tiller density at either marsh. D) Effect of press (solid line) and pulse (dashed line) treatments on grams of nitrogen per square meter of marsh surface in live *Spartina* biomass. E) Effect of press (solid line) and pulse (dashed line) treatments on grams of *Spartina* thatch per square meter.
(TIF)

Figure S2 **Effects of press and pulse treatments on additional herbivores and algivores not included in** **Figure 1** **.** Effects for press (solid line) and pulse (dashed line) treatments are displayed for Cape Hatteras National Seashore, NC (panels on left), and Tuckerton, NJ (panels on right). Error bars indicate standard errors of the means. Effect means within gray bands were not significantly different from zero, meaning that treatment and control values did not differ. Asterisks indicate that pulse and press treatments had significantly different effects in a given year (alpha  = 0.05). Treatment effect was calculated as ln((treatment value +1)/(control value +1)), where “treatment value” was the value from the press or pulse plot in a block, and “control value” was the value from the control plot in that same block. A) Effect of press (solid line) and pulse (dashed line) treatments on the density of the planthopper *Delphacodes penedetecta*. The pre-treatment press effect at Cape Hatteras was not significant despite its high mean because of high variance among blocks. B) Effect of press (solid line) and pulse (dashed line) treatments on the density of the mirid herbivore *Trigonotylus uhleri*. C) Effect of press (solid line) and pulse (dashed line) treatments on the density of the amphipod *Orchestia grillus*. Very few amphipods were collected at the Tuckerton marsh. D) Effect of press (solid line) and pulse (dashed line) treatments on the density of the isopod *Venezillo parvus*. Virtually no isopods were collected at Cape Hatteras.
(TIF)

Figure S3 **Effects of press and pulse treatments on additional omnivore and predator densities not included in** **Figure 1** **.** Effects for press (solid line) and pulse (dashed line) treatments are displayed for Cape Hatteras National Seashore, NC (panels on left), and Tuckerton, NJ (panels on right). Error bars indicate standard errors of the means. Effect means within gray bands were not significantly different from zero, meaning that treatment and control values did not differ. Asterisks indicate that pulse and press treatments had significantly different effects in a given year (alpha = 0.05). Treatment effect was calculated as ln((treatment value +1)/(control value +1)), where “treatment value” was the value from the press or pulse plot in a block, and “control value” was the value from the control plot in that same block. A) Effect of press (solid line) and pulse (dashed line) treatments on densities of katydids per square meter. Katydids belonged to the genera *Conocephalus* and *Orchelimum*. Katydid densities at Tuckerton were too low to calculate reliable treatment effects. B) Effect of press (solid line) and pulse (dashed line) treatments on densities of web-building spiders per square meter. The most common families of web-building spiders at both marshes were Linyphiidae and Dictynidae. C) Effect of press (solid line) and pulse (dashed line) treatments on densities of hunting spiders other than *Pardosa* per square meter. Effects of fertilization on *Pardosa* density are displayed in figures 1I and 1J. During the pre-treatment collection, hunting spiders were found in only three blocks at Tuckerton.
(TIF)

Figure S4 **Effects of fertilization treatment on degree of herbivore damage to** ***Spartina*** **plants at CHNS.** A) Fraction of leaf with damage caused by katydids (*Conocephalus spartinae* and *Orchelimum fidicinium*). B) Fraction of leaf with damage caused by the herbivorous mirid *Trigonotylus uhleri*.
(TIF)

Table S1 Compilation of dates that experimental manipulations were initiated or maintained and that plant and arthropod samples were collected. At both of our field sites (Tuckerton, NJ [TUCK] and Cape Hatteras National Seashore, NC [CHNS]), fertilization treatments were initiated in 2005 and maintained from 2006–2008. Below we list the dates that we fertilized treatment plots and collected samples to measure plant biomass, plant %N (both within and 1 m outside study plots) and arthropod abundance.
(DOC)
