# Supplementary material for: Nutrient Presses and Pulses Differentially Impact Plants, Herbivores, Detritivores and Their Natural Enemies
Source: PLoS One. 2012 Aug 28;7(8):e43929. doi: 10.1371/journal.pone.0043929 (PMC3429447; doi:10.1371/journal.pone.0043929)
Supplement: Table S1 — Compilation of dates that experimental manipulations were initiated or maintained and that plant and arthropod samples were collected. At both of our field sites (Tuckerton, NJ [TUCK] and Cape Hatteras National Seashore, NC [CHNS]), fertilization treatments were initiated in 2005 and maintained from 2006–2008. Below we list the dates that we fertilized treatment plots and collected samples to measure plant biomass, plant %N (both within and 1 m outside study plots) and arthropod abundance. (DOC) [file pone.0043929.s005.doc]

**Table S1.** Compilation of dates that experimental manipulations were initiated or maintained and that plant and arthropod samples were collected. At both of our field sites (Tuckerton, NJ [TUCK] and Cape Hatteras National Seashore, NC [CHNS]), fertilization treatments were initiated in 2005 and maintained from 2006-2008. Below we list the dates that we fertilized treatment plots and collected samples to measure plant biomass, plant %N (both within and 1m outside study plots) and arthropod abundance.

**Site Year Dates Fertilized Plant Biomass Plant %N Plant %N Arthropods**

**1m outside plot**

TUCK 2005 5/14, 6/5, 6/27 5/14, 7/19, 10/3 5/14, 7/12, 7/19, 10/3 7/19 5/14, 7/12, 8/17, 9/7, 10/3

2006 5/17, 6/6, 6/28 8/31 5/17, 7/25, 8/31 7/25 5/17, 6/28, 7/25, 8/31, 10/12

2007 5/17, 5/30, 6/21 7/19 5/17, 6/28, 7/19 5/17, 6/21, 7/17, 8/25

2008 5/22, 6/17, 7/20 8/14 5/22, 7/20, 8/14 5/22, 6/17, 7/20, 8/14

CHNS 2005 5/16, 6/8, 7/6 5/16, 8/2, 9/28 5/16, 8/2, 9/28 8/2 5/16, 6/8, 7/6, 8/2, 9/28

2006 5/2, 6/8, 7/6 8/17 5/2, 7/6, 8/17 8/17 5/2, 6/8, 7/6, 8/17, 10/3

2007 5/11, 6/8, 7/9 8/22 5/11, 7/9, 8/22 5/11, 6/8, 7/9, 8/22

2008 5/13, 6/13, 7/12 8/9 5/13, 7/12, 8/9 5/13, 6/13, 7/12, 8/9
